# Phase 1 study of high-dose DFMO, celecoxib, cyclophosphamide and topotecan for patients with relapsed neuroblastoma: a New Approaches to Neuroblastoma Therapy trial

**DOI:** 10.1038/s41416-023-02525-2

**Published:** 2024-01-10

**Authors:** Michael D. Hogarty, David S. Ziegler, Andrea Franson, Yueh-Yun Chi, Denice Tsao-Wei, Kangning Liu, Rohan Vemu, Eugene W. Gerner, Elizabeth Bruckheimer, Anasheh Shamirian, Beth Hasenauer, Frank M. Balis, Susan Groshen, Murray D. Norris, Michelle Haber, Julie R. Park, Katherine K. Matthay, Araz Marachelian

**Affiliations:** 1 Division of Oncology, The Children’s Hospital of Philadelphia, Philadelphia, PA USA; 2 Department of Pediatrics, Perelman School of Medicine at the University of Pennsylvania, Philadelphia, PA USA; 3 Children’s Cancer Institute, Lowy Cancer Research Centre, Randwick, NSW Australia; 4 School of Women’s and Children’s Health, University of New South Wales, Sydney, Australia; 5 Kids Cancer Centre, Sydney Children’s Hospital, Randwick, NSW Australia; 6 Department of Pediatrics, University of Michigan Medical School, Ann Arbor, MI USA; 7 Children’s Hospital Los Angeles, University of Southern California Keck School of Medicine, Los Angeles, CA USA; 8 Department of Preventive Medicine, University of Southern California, Los Angeles, CA USA; 9 Cancer Prevention Pharmaceuticals, Tucson, AZ USA; 10 Panbela Pharmaceuticals, Inc., Waconia, MN USA; 11 St. Jude Children’s Research Hospital, University of Tennessee, Memphis, TN USA; 12 UCSF Benioff Children’s Hospital, UCSF School of Medicine, University of California San Francisco, San Francisco, CA USA

**Keywords:** Targeted therapies, Embryonal neoplasms

## Abstract

**Background:**

*MYC* genes regulate ornithine decarboxylase (Odc) to increase intratumoral polyamines. We conducted a Phase I trial [NCT02030964] to determine the maximum tolerated dose (MTD) of DFMO, an Odc inhibitor, with celecoxib, cyclophosphamide and topotecan.

**Methods:**

Patients 2–30 years of age with relapsed/refractory high-risk neuroblastoma received oral DFMO at doses up to 9000 mg/m^2^/day, with celecoxib (500 mg/m^2^ daily), cyclophosphamide (250 mg/m^2^/day) and topotecan (0.75 mg/m^2^/day) IV for 5 days, for up to one year with G-CSF support.

**Results:**

Twenty-four patients (median age, 6.8 years) received 136 courses. Slow platelet recovery with 21-day courses (dose-levels 1 and 2) led to subsequent dose-levels using 28-day courses (dose-levels 2a-4a). There were three course-1 dose-limiting toxicities (DLTs; hematologic; anorexia; transaminases), and 23 serious adverse events (78% fever-related). Five patients (21%) completed 1-year of therapy. Nine stopped for PD, 2 for DLT, 8 by choice. Best overall response included two PR and four MR. Median time-to-progression was 19.8 months, and 3 patients remained progression-free at >4 years without receiving additional therapy. The MTD of DFMO with this regimen was 6750 mg/m^2^/day.

**Conclusion:**

High-dose DFMO is tolerable when added to chemotherapy in heavily pre-treated patients. A randomized Phase 2 trial of DFMO added to chemoimmunotherapy is ongoing [NCT03794349].

## Background

Children with relapsed or refractory neuroblastoma have dismal outcomes [1] and novel treatments are sought. Amplification of the *MYCN* gene is frequent [2] and is correlated with treatment failure [3]. Tumors that lack *MYCN* amplification often have alternative *MYCN* or *MYC* deregulation [4–6] so hyperactivated Myc is the principal oncogenic driver. While directly targeting Myc has proven difficult, pathways transcriptionally regulated by Myc may be druggable. Myc genes redirect metabolism to promote the synthesis of nucleic acids, proteins, lipids and polyamines [7]. Polyamines are oncometabolites essential for *MYC*s tumor-promoting effects [8], polyamine enzymes are coordinately dysregulated in neuroblastomas [9, 10], and therapeutics that antagonize polyamine homeostasis show anti-tumor activity across complementary preclinical neuroblastoma models [9, 11, 12].

In this Phase 1 trial, we sought to deplete intra-tumoral polyamines by both inhibiting their synthesis and augmenting their export. DFMO (α-difluoromethylornithine; Eflornithine) is a covalent inhibitor of ornithine decarboxylase (Odc), an enzyme transcriptionally regulated by *MYC* genes that is rate-limiting for polyamine biosynthesis. Celecoxib is a cyclo-oxygenase-2 inhibitor that upregulates spermidine/spermine-N1-acetyltransferase (encoded by *SAT1*) that augments polyamine export from cells. This combination was given with cyclophosphamide and topotecan, a chemotherapy regimen with tolerability and activity in heavily pre-treated neuroblastoma patients [13].

DFMO is FDA-approved for the treatment of *Trypanosomiasis*, as Odc is an essential trypanosome gene [14]. Doses range from 12,000–18,000 mg/m^2^/day IV and toxicities are primarily gastrointestinal [15]. Since polyamines are essential for human cell proliferation and are abundant in tumor cells, DFMO has been explored as a cancer agent at doses up to 21,000 mg/m^2^/day [16]. Chronic oral administration in cancer settings has had a similar toxicity profile, in addition to modest reversible hearing loss across frequencies that can be mitigated with intermittent dosing [17]. The MTD of oral DFMO combined with chemotherapy in adult carcinoma trials is 9000 mg/m^2^/day [18]. Anti-tumor activity has been limited but no genomic selection strategy has been employed to enrich for polyamine dependent tumors. Evidence links *MYC* hyperactivity to polyamine addiction and tumors like neuroblastoma may be particularly vulnerable to polyamine depletion therapeutics [9, 10, 12, 19].

DFMO has also been studied at lower doses from 500 to 1500 mg/m^2^/day for cancer chemoprevention in adults [20, 21] and for minimal residual disease treatment in children with neuroblastoma [22, 23]; and at up to 3000 mg/m^2^/day with low-dose oral etoposide for relapsed neuroblastoma patients [24]. However, no anti-tumor activity is seen in preclinical models at systemic DFMO exposures corresponding to these doses, likely due to insufficient depletion of intracellular polyamines. Here we report the first study of high dose DFMO, intended to maximize Odc inhibition in Myc hyperactivated tumors, in concert with celecoxib to further enhance polyamine depletion. Our objective was to define the MTD of DFMO (with a 7-day break to minimize ototoxicity) with celecoxib, cyclophosphamide and topotecan in patients with advanced neuroblastoma. We assessed DFMO PK for comparison with plasma concentrations achieved in preclinical mouse models that demonstrate anti-neuroblastoma efficacy, *ODC1* functional polymorphisms [25–27], and preliminary anti-tumor activity for this combination.

## Patients and methods

### Patients

Patients 2–30 years of age with relapsed or refractory high-risk neuroblastoma, with evaluable disease by bone marrow morphology, imaging (soft tissue lesion ≥10 mm, or ≥15 mm for tumor-involved lymph nodes), or metaiodobenzylguanidine (MIBG) scans obtained within 4 weeks of enrollment, were eligible. Patients were classified as having relapse or progression, or being refractory to initial therapy (less than partial response [PR] by International Neuroblastoma Response Criteria (INRC; [28]) after ≥4 cycles of chemotherapy; or PR after ≥4 cycles of chemotherapy with biopsy-confirmed persistent disease).

Patients were a minimum of 3 weeks from systemic therapy, 12 weeks from myeloablative therapy, 2 weeks from small-port radiation, 6 weeks from iodine^131^-MIBG therapy or substantive marrow-involved radiation therapy, and 6 months from total body or larger-field radiation. Patients previously treated with cyclophosphamide and topotecan were eligible if they did not have disease progression while being treated. Prior DFMO exposure was not a criterion for exclusion.

All patients met standard organ function criteria before enrolling. Patients were excluded if they were pregnant, breastfeeding, had undergone allogeneic stem-cell transplantation, required hemodialysis, or had an active infection. DFMO-specific exclusions included CNS parenchymal or meningeal disease, a seizure within 12 months, or need for anticonvulsant medications for seizure control. Celecoxib-specific exclusions included active gastrointestinal bleeding, symptomatic gastritis, or aspirin/NSAID hypersensitivity. Patients and/or legal guardians provided written informed consent. The institutional review board of each New Approaches to Neuroblastoma Therapy (NANT) site approved the study.

### Protocol therapy

Patients received cyclophosphamide (250 mg/m^2^) followed by topotecan (0.75 mg/m^2^) intravenously over 30 minutes for 5 consecutive days each course. Granulocyte colony stimulating factor was initiated after chemotherapy and continued through blood count recovery (or one dose for pegylated-G-CSF). Celecoxib was given orally at 250 mg/m^2^ twice daily for patients with a body surface area (BSA) ≥0.40 m^2^, or 500 mg/m^2^ daily for those with a BSA < 0.40 m^2^. Powder-formulated DFMO (eflornithine hydrochloride) was reconstituted with sterile water to a 100 mg/ml solution and given orally three times daily. For dose levels 1 and 2 (21-day courses), chemotherapy was days 1–5 and DFMO was given for 14 days out of 21 (held for days 8–14). Due to delayed platelet recovery, subsequent dose levels 2a-4a used 28-day courses, with DFMO given days 1–14 and 22–28, chemotherapy days 8–12 (the same regimen as dose levels 1 and 2 but with 7 additional days of DFMO to begin each cycle). All courses incorporated a 7-day DFMO break to minimize ototoxicity and included continual celecoxib.

Patients were eligible to receive 1-year of therapy in the absence of disease progression: 17 courses for dose levels 1 and 2 (21-day courses) or 12 courses for dose levels 2a-4a (28-day courses). For a first hematologic DLT, patients on dose level 1 had chemotherapy doses reduced by 20%, and if hematologic DLT recurred, the patient discontinued protocol therapy. For patients on dose levels ≥2, DFMO was reduced to the next lower dose level, and if hematologic toxicity recurred, cyclophosphamide and topotecan doses were reduced 20%. If hematologic DLT recurred, the patient discontinued protocol therapy. For diarrhea DLT, DFMO was held while awaiting evaluation for an infectious cause and resumed at the same dose if an infection was identified, or the next lower DFMO dose level for no infectious cause once diarrhea resolved to grade ≤1. For all other DLT, patients had DFMO reduced to the next lower dose level (or discontinued protocol therapy if on dose level 1). For a subsequent DLT, patients discontinued protocol therapy. Celecoxib was held for any platelet count <20,000/mm^3^ and resumed when the platelet count was above this, with or without platelet transfusion. Celecoxib was also held 14 days for gastritis DLT and resumed at 50% dosing if symptoms resolved, with increases to full dose if tolerated with gastric protection (proton pump inhibitor and sucralfate use); and discontinued for grade ≥3 GI bleeding.

### Toxicity assessment

Toxicity was graded according to the Common Terminology Criteria for Adverse Events, version 4.0. Patients who received <80% of prescribed DFMO, celecoxib, cyclophosphamide or topotecan during course 1, in the absence of DLT, were replaced for dose escalation decisions. Hematologic DLT was defined as a >14-day delay in the start of a subsequent course due to neutropenia (<750/mm^3^) or thrombocytopenia (<75,000/mm^3^). Non-hematologic DLT was defined as any non-hematologic toxicity that delayed the start of a subsequent course by more than 14 days, or any grade ≥3 toxicity with the exception of nausea, vomiting, anorexia, or dehydration resolving to grade ≤2 within 72 h; increase in transaminase or electrolyte abnormality resolving to grade ≤1 within 7 days; diarrhea persisting <72 h; hematuria resolving to grade ≤1 within 7 days; and fever; infection; or febrile neutropenia. Since reversible ototoxicity was observed in adult DFMO trials and most children enrolled had hearing loss vulnerability from prior cisplatin exposure, hearing was a targeted toxicity. DLT included any hearing loss from baseline of >15 dB at two contiguous frequencies between 500 and 3000 Hz that did not return to pre-therapy baseline within 3 weeks.

### Response evaluation

Patients underwent disease evaluation at baseline, after courses 2, 4, and 8, and at the completion of therapy. Response was graded according to the NANT Response Criteria (v1.2), a modification of the INRC19 [28] described previously [29], and uses Response Evaluation Criteria in Solid Tumors (RECIST) for measurable tumors, Curie score for MIBG scan response, and BM morphology. Minor response (MR) includes CR or PR in at least one component and no component with PD. Patients with SD or better overall responses underwent central review to confirm. Objective responses included overall responses of CR or PR. Patients who received <80% of study drugs in course 1 in the absence of PD or DLT were deemed inevaluable for response.

### Pharmacokinetics and *ODC1* SNP studies

Three mL of blood were drawn prior to the next scheduled DFMO dose on days 1 and 5 of chemotherapy in courses 1 and 2 to obtain steady-state DFMO trough levels. Samples were centrifuged and plasma extracted and frozen at −80 °C. To compare DFMO PK parameters with those achieved in murine models, *TH-MYCN*^*+/+*^ mice [30] were randomized by litter at the time of weaning, and provided ad libitum access to water with DFMO added at 0.25%, 0.5%, 1.0% or 1.5% (*n* = 4–6 each) as in preclinical studies [9, 11, 12]. Blood was drawn after 5-7 days at the end of a 12-h sleep cycle (to mimic a trough level) and processed as above. Mouse work was done under an IACUC approved protocol at the Children’s Hospital of Philadelphia. DFMO concentration was measured using an HPLC MS/MS assay with a validated calibration range from 50–100,000 ng/ml (InVentiv Health, Inc., Quebec, Canada). The study included an optional pharmacogenomics aim to genotype functional *ODC1* promoter polymorphisms. Consenting patients provided 3 ml of whole blood in EDTA tubes at any time on study. After extracting DNA with a QIAamp DNA Blood Mini Kit (Qiagen, Santa Clarita, CA), *ODC1* polymorphism genotyping for SNPs rs2302615 (G316A) and rs2302616 (G263T) were performed using primers and probes from ThermoFisher (Waltham, MA).

### Statistical methods

Evaluation of DFMO dose levels followed the 3 + 3 dose escalation design [31]. Only DLTs in the first course of therapy had an impact on decisions regarding dose escalation. Patients were evaluable for DLT if they had a DLT during the first course, or if they completed the first course of therapy without DLT and received ≥80% of prescribed study therapy. The MTD was the highest dose level tested at which fewer than two of six patients had first course DLT. Progression-free survival (PFS) was estimated by using Kaplan–Meier methods as time from the start of treatment to first episode of disease progression or death; patients who were alive and without progression were censored at last follow-up or start of new therapy. The means and 95% confidence intervals for trough DFMO PK levels were calculated for patients with known time from prior DFMO dose based on a generalized linear model with dose level and time from prior DFMO dose as fixed effects and patient as random effect. Geometric means were calculated means using log-transformed trough levels. A one-compartment population pharmacokinetic model was fit to the data using Phoenix NLME module (Certara, Princeton, NJ). Analyses were performed with STATA version 11 (STATA, College Station, TX), SAS version 9.4 (Cary, NC), and R version 4.0.2.

## Results

### Patient characteristics

Twenty-four patients were enrolled from January 2014 to June 2017, and no patient was deemed ineligible after enrollment. Patient characteristics are provided in Table 1. All 24 (100%) had stage 4 (or M) neuroblastoma at original diagnosis, and seven of 20 patients (35%) with available data had *MYCN* amplified tumors. Twenty-one (88%) had recurrent or progressive disease, 23 (96%) had MIBG avid disease, and 12 (50%) had bone marrow involvement at the time of enrollment. Twenty patients (83%) previously received cyclophosphamide and topotecan, no patient previously received DFMO; 21 (88%) had undergone prior autologous stem cell transplant and 5 (21%) previously received chemoimmunotherapy with anti-GD2 antibody. All patients were evaluable for dose escalation decisions; one patient missed 16 of 56 celecoxib doses in course 1 and was inevaluable for response evaluation but had a DLT so was included for toxicity assessment.

**Table 1:** Characteristics of enrolled and eligible patients (*n* = 24).

Patient and disease characteristics	Value
Median age at study entry (range), years	6.8 (2.0–19.6)
Median time from diagnosis to study entry (range), months	31 (9–89)
Sex, *n* (%)	
Female	9 (38)
Male	15 (62)
Stage 4 at initial diagnosis, *n* (%)	24 (100)
Disease status at study enrollment, *n* (%)	
Refractory disease	3 (12)
Recurrent/progressive disease	21 (88)
Tumor *MYCN* status, *n* (%)	
Amplified	7 (29)
Not amplified	13 (54)
Unknown	4 (17)
Disease at study enrollment, *n* (%)	
Soft tissue involvement	15 (62)
MIBG avid disease	23 (96)
• Median Total Absolute Curie Score (range)	5 (1–21)
Bone Marrow involvement	12 (50)
Therapy received prior to enrollment, *n* (%)	
Prior DFMO	0 (0)
Prior cyclophosphamide/topotecan	20 (83)
Prior stem cell transplant	21 (88)
Prior chemoimmunotherapy	5 (21)

### Dose escalation and toxicity

Dose escalation details are provided in Table 2. Overall, there were three first course DLTs (1 at dose level 3a, 2 at dose level 4a) and two subsequent course DLTs (both at dose level 1). The study defined maximal tolerated dose and recommended Phase 2 dose for DFMO was 6750 mg/m^2^/day. By dose level: three patients each were treated at dose levels 1 and 2 without a first course DLT; two patients on dose level 1 had a subsequent DLT (one hematuria with concurrent BK virus infection in course 2, and one grade 3 hypotension in course 11). Both came off study as no allowance was made to reduce DFMO dose below dose level 1. Delayed platelet recovery that did not meet DLT criteria, but delayed therapy, led the study committee to change from 21-day to 28-day courses following dose level 2 (called dose levels 2a, 3a and 4a). This decreased the relative dose-intensity for cyclophosphamide and topotecan by 25% (from 5 of 21 days to 5 of 28 days) while increasing the dose-intensity of DFMO by 12% (from 14 of 21 days to 21 of 28 days).

**Table 2:** Dose escalation of DFMO in combination with fixed doses of celecoxib, cyclophosphamide and topotecan.

Dose level	Difluoromethylornithine (mg/m^2^ per day)^a^	No. of patients entered	No. eligible and evaluable for DLT	No. evaluable with DLT in first course
1	3000	3	3	0
2	4500	3	3	0
2a	4500	6	6^b^	0
3a	6750	6	6	1^c^
4a	9000	6	6	2^d^

^a^See text for schedules for dose levels 1-2 versus 2a-4a.

^b^No DLTs during course 1, however, the first three patients had delay in blood count recovery, so the dose level was expanded to enroll three additional patients.

^c^Grade 4 platelet count decrease.

^d^Grade 3 anorexia and grade 4 alanine aminotransferase increase.

No first course DLT occurred in 3 patients treated at dose level 2a, yet two had delay in platelet recovery, so 3 additional patients were enrolled and were without DLT or platelet delay. One of three patients on dose level 3a had a first course DLT for cytopenias. This patient had progressive disease with new bone lesions, pleural effusion, and grade 2 somnolence and hallucinations. Intra-tumoral bleeding was seen on imaging but there was no evidence for disseminated intravascular coagulation or bone marrow involvement, leading to a DLT determination (>14-day delay in platelet count recovery). No additional DLT occurred in three additional patients on dose level 3a. Six total patients were treated at dose level 4a and 2 experienced a first course DLT. One with pre-existing poor nutrition was admitted for febrile neutropenia and grade 2 mucositis, and nasogastric tube feeds were initiated, meeting criteria for DLT (grade 3 anorexia >72 h). The second started therapy with grade 1 ALT/AST elevation that progressed to grade 3 following 10 doses of DFMO and did not resolve to grade 1 after 7 days of DFMO discontinuation.

The 24 eligible patients received a total of 136 courses of therapy (range 1–17). Hematologic toxicity was common in this heavily pre-treated population (Table 3), with grade 3 or 4 thrombocytopenia, leukopenia or neutropenia in 92%, 74%, and 68% of courses, respectively. Twenty febrile neutropenia events occurred in 10 patients, associated with PCR-confirmed viral respiratory infection in three patients. Additional infections included 1 *Klebsiella* UTI (with hematuria and hypotension), 1 gastric tube-associated cellulitis (grade 2), 1 BK virus UTI (with hematuria and viremia), and 2 central line-associated bacterial infections. Grade 1 or 2 fatigue, nausea and emesis were reported for >25% of courses. Grade 3 or 4 hepatic toxicity occurred in three patients (13%) and consisted of grade 3 elevation of ALT and/or AST in 3 (1 with elevations in multiple courses, including a grade 4 elevation that defined a DLT). Two patients with liver enzyme elevations, including the patient with DLT, were receiving DFMO at dose level 4a.

**Table 3:** Percent of hematologic and non-hematologic toxicities observed in courses from evaluable patients according to dose level and grade.

Toxicity	Any grade toxicity (136 courses in 24 patients), % courses (# of patients)	Dose level 1 (17 courses in 3 patients)				Dose level 2 (34 courses in 3 patients)				Dose level 2a (21 courses in 6 patients)				Dose level 3a (27 courses in 6 patients)				Dose level 4a (37 courses in 6 patients)			
		1	2	3	4	1	2	3	4	1	2	3	4	1	2	3	4	1	2	3	4
Hematologic toxicities																					
Anemia	97 (24)	0	24	76	0	0	15	85	0	0	24	62	0	4	19	74	4	0	24	73	0
Platelet count decreased	96 (24)	0	0	6	94	0	6	68	26	0	10	10	71	0	7	22	70	0	0	5	86
White blood cell decreased	90 (24)	0	0	0	100	21	21	29	26	0	5	5	33	7	4	4	85	0	11	3	84
Neutrophil count decreased	77 (22)	0	6	6	82	0	12	24	15	5	5	0	48	4	4	4	85	3	5	5	78
Lymphocyte count decreased	65 (18)	0	6	29	0	6	3	29	12	0	0	5	5	0	4	7	89	0	3	57	38
Lymphocyte count increased	1 (1)	0	0	0	0	0	0	0	0	0	0	5	0	0	0	0	0	0	0	0	0
Non-hematologic toxicities																					
Nausea	43 (20)	35	0	0	0	82	0	0	0	24	14	0	0	15	0	0	0	24	5	3	0
Vomiting	31 (16)	24	0	0	0	32	0	0	0	14	5	5	0	15	0	0	0	41	5	3	0
Aspartate aminotransferase increased	27 (9)	18	0	0	0	0	0	0	0	29	10	5	0	4	0	0	0	49	8	8	0
Alanine aminotransferase increased	24 (11)	0	12	0	0	3	0	0	0	10	0	5	0	15	0	0	0	11	27	19	5
Fatigue	23 (14)	24	0	0	0	3	0	0	0	10	0	0	0	19	4	0	0	43	5	0	0
Diarrhea	18 (15)	6	0	0	0	6	0	0	0	14	10	0	0	33	4	4	0	11	3	3	0
Anorexia	18 (13)	18	0	0	0	3	6	0	0	5	0	0	0	4	22	0	0	22	3	3	0
Mucositis oral	17 (5)	0	0	0	0	0	0	0	0	0	0	0	0	15	4	0	0	35	14	0	0
Alopecia	15 (4)	0	0	0	0	38	3	0	0	0	0	0	0	4	7	0	0	0	11	0	0
Febrile neutropenia	12 (10)	0	0	29	0	0	0	0	0	0	0	10	0	0	0	22	0	0	0	11	0
Abdominal pain	12 (9)	29	0	0	0	18	0	0	0	0	0	0	0	7	0	0	0	11	0	0	0
Alkaline phosphatase increased	12 (3)	12	0	0	0	0	0	0	0	10	14	0	0	0	0	0	0	24	0	0	0
Epistaxis	10 (7)	12	0	0	0	3	0	0	0	0	0	0	0	15	4	0	0	14	0	0	0
Bruising	10 (3)	0	0	0	0	0	0	0	0	0	0	0	0	4	0	0	0	11	22	0	0
GGT increased	9 (4)	18	0	0	0	0	0	0	0	0	0	0	0	0	0	0	0	24	0	0	0
Headache	8 (9)	6	0	0	0	3	3	0	0	14	0	0	0	7	0	0	0	5	3	0	0
Weight loss	8 (3)	0	0	0	0	0	12	0	0	0	0	0	0	7	19	0	0	0	0	0	0
Hypoalbuminemia	7 (9)	6	0	0	0	0	0	0	0	5	0	0	0	19	0	0	0	8	0	0	0
Fever	6 (7)	12	0	0	0	0	0	0	0	14	0	0	0	0	4	0	0	5	0	0	0
Hematuria	6 (4)	0	0	12	0	9	0	0	0	0	0	0	0	11	0	0	0	0	0	0	0
Hypophosphatemia	5 (5)	0	0	0	0	0	0	0	0	5	0	0	0	15	0	0	0	5	0	0	0
Allergic reaction	4 (2)	0	0	0	0	0	0	0	0	0	0	0	0	0	0	0	0	3	14	0	0
Flatulence	4 (2)	0	0	0	0	0	0	0	0	0	0	0	0	19	4	0	0	0	0	0	0
Hypermagnesemia	3 (2)	0	0	0	0	0	0	0	0	0	0	0	0	15	0	0	0	0	0	0	0
Rectal pain	3 (2)	12	12	0	0	0	0	0	0	0	0	0	0	0	0	0	0	0	0	0	0

Percentage of affected courses shown for all courses of therapy. Only toxicities occurring in greater than 10% of courses in at least one dose level are shown. Toxicities attributed as unrelated/unlikely to protocol therapy are not shown.

Ototoxicity was a targeted toxicity based on prior experience with high DFMO. Audiograms were obtained after courses 2, 4, 8, 12 and at treatment completion. Grade 3 hearing loss occurred in 1 patient (4%) on dose level 2 and hearing normalized at re-testing and DFMO therapy was resumed without recurrent ototoxicity. DFMO was not associated with worsening of high-frequency hearing loss in patients with prior cisplatin toxicity. Any grade diarrhea occurred in 18% of courses and was correlated with dose level, as seen in adult trials [32], but grade 3 or 4 diarrhea was seen in only 2 courses (1 each at dose level 3a and 4a).

### DFMO pharmacokinetics

Eighty DFMO levels were obtained from 23 study patients. DFMO trough levels (mean ± standard deviation) from patients on dose levels 1, 2, 2a, 3a, and 4a were 12.3 ± 9.7 µmol/L, 53.2 ± 32.7 µmol/L, 55.6 ± 54.1 µmol/L, 73.9 ± 59.9 µmol/L, and 84.4 ± 61.4 µmol/L, respectively. There was marked intra- and inter-patient variability and time from prior DFMO dose to blood sampling varied widely (range, 1–17 h). To control for this, we repeated our analyses using 49 samples from the 18 patients on dose levels 2a-4a in which time from prior DFMO dose was documented. Accounting for time, trough concentrations [geometric mean (95% CI)] were 48.2 (29.3–79.4) µmol/L, 65.8 (40.6–106.7) µmol/L, and 72.9 (42.8–124.1) µmol/L, respectively, with significant residual variability that was not attributable to DFMO dose timing. A one-compartment population pharmacokinetic model was fit to these data. Parameter values modeled from time-defined DFMO PK included: k_a_ (absorption rate constant) of 0.0728 ± 0.0285/h; V (volume of distribution) of 10.13 ± 6.01 L/h/m^2^; and k_el_ (elimination rate constant) of 0.635 ± 0.312/h.

DFMO has anti-tumor activity as a single agent and when combined with chemotherapy in mouse models of neuroblastoma [9, 10, 12, 19], including the *TH-MYCN* neuroblastoma-prone mouse [30]. Extended survival is recurrently demonstrated for mice taking 1% DFMO ad libitum, so *TH-MYCN*^*+/+*^ mice were provided water ad libitum with 0.25, 0.5, 1.0 or 1.5% DFMO for 5-7 days and plasma was collected following their sleep period to mimic a trough DFMO level. DFMO concentrations (mean ± standard deviation) were 29.7 ± 14.1 µmol/L, 56 ± 31.8 µmol/L, 53.8 ± 49.5 µmol/L, and 60.9 ± 46.5 µmol/L, respectively across these DFMO exposures, also with significant between mouse variability.

### *ODC1* promoter polymorphisms

The *ODC1* gene contains SNPs that may influence transcriptional response to Myc, risk for carcinogenesis [26] and response to therapeutic interventions [24, 27]. We genotyped the G316A (rs2302615) and G263T (rs2302616) SNPs in 17 patients. For the G316A SNP, 15 patients were homozygous for the major allele (G/G), two were heterozygous (G/A). For the G263T SNP, 6 patients were homozygous for the major allele (G/G), ten were heterozygous (G/T), and one was homozygous for the minor allele (T/T). SNP genotypes, *MYCN* amplification and clinical metrics are provided in Supplementary Table S1. There was no correlation identified among clinical response, toxicity, and genomic marker; although the G316A risk allele (G) was overrepresented in our cohort: 0.941 allele frequency, relative to 1000Genomes (American) allele frequency of 0.748.

### Anti-tumor activity

**Fig. 1:**
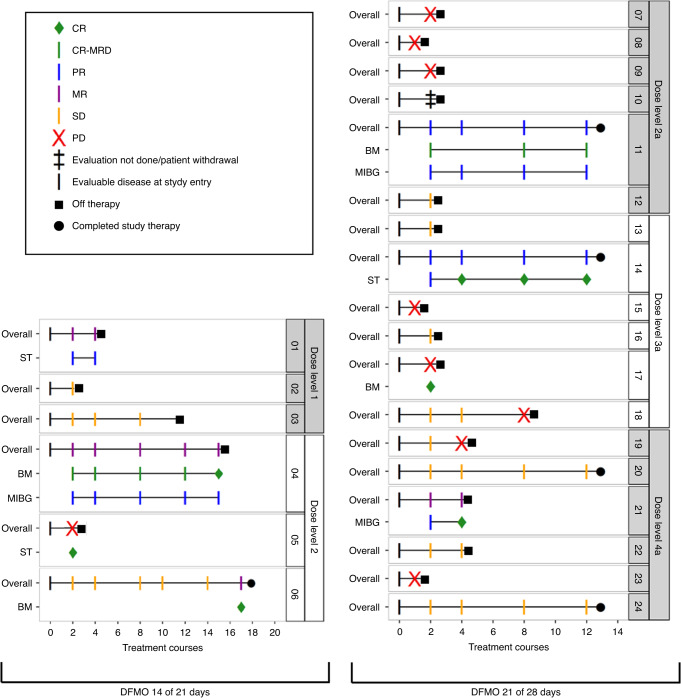
Swimmers Plot of all patients enrolled (*n* = 24). Overall (all responses shown) and component ST, BM and MIBG responses (only CR and PR shown) in enrolled patients, by dose level (dose levels 1–2, 21 day cycles, on left; dose levels 2a-4a, 28 day cycles, on right). CR complete response, CR-MRD complete response-minimal residual disease, MR minor response, PD progressive disease, PR partial response, SD stable disease, ST soft tissue, BM bone marrow, MIBG metaiodobenzylguanethidine detected disease.

**Fig. 2:**
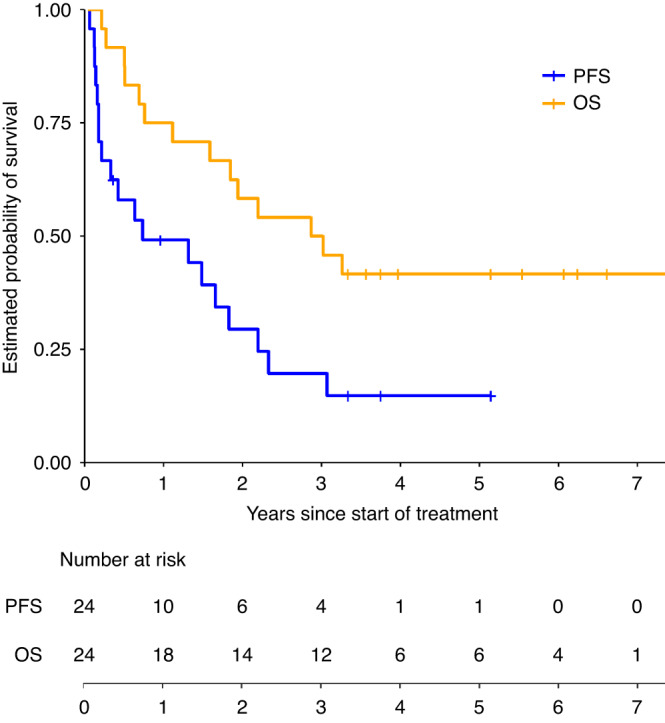
Kaplan–Meier plots for progression-free survival and overall survival for all patients (*n* = 24). At 2 years after starting treatment, PFS probability is 0.295 (95% CI [0.153, 0.566]) and OS probability is 0.583 (95% CI [0.416, 0.818]). For PFS, patients without PD were censored at start of new treatment, or last follow-up if no new treatment. PFS progression-free survival, OS overall survival.

Five patients (21%) completed 1-year of therapy without disease progression (2 with PR, 1 MR, 2 SD); 9 stopped therapy due to PD, 2 stopped due to DLT (DFMO dose level 1 without dose de-escalation option), and 8 stopped by choice after 2–15 courses. Best overall response included 2 PR, 4 MR, 10 SD, 7 PD and 1 inevaluable (Fig. 1 and Table 4). All patients with an overall response of PR or MR sustained this response until stopping or completing protocol therapy. The overall objective response rate (CR + PR) was 9% (95% CI, 1.5–29.5%) and rate of any response (CR + PR + MR) was 26%. INRC component-CRs included bone marrow CR in 4 patients, soft tissue lesion CR in 2, and MIBG avid metastatic site CR in 1. Responses included patients with *MYCN* amplified and non-amplified tumors. All patients that responded had received prior cyclophosphamide and topotecan chemotherapy. At 2 years, PFS for the entire cohort was 29.5% (95% CI, 15.3–56.6%) and OS was 58.3% (95% CI, 41.6–81.8%; Fig. 2). Notably, three patients completed protocol therapy and remain without disease progression or event at >4 years from treatment end in the absence of additional therapy. One was originally treated for intermediate-risk stage M disease at 10 months of age, then relapsed at 3 years of age with diffuse metastatic disease (11q- and 17q+ segmental chromosome alterations). Following high-risk therapy that included autologous stem cell rescue, GD2 immunotherapy, and radiotherapy, the patient relapsed with facial bone and soft tissue disease in an irradiated field, enrolled on this trial and is progression free at 4.9 years from treatment end. A second patient was diagnosed with stage M disease at 3.8 years of age and progressed following high-risk therapy, MIBG therapy, salvage chemotherapy, and GD2 immunotherapy. This patient completed DFMO therapy 4.9 years ago and remains in CR. A third patient was treated for infant stage M disease that progressed to high-risk metastatic disease (diploid tumor, 11q- and 17q+ segmental chromosome alterations). The patient was refractory to high-risk therapy and enrolled on this trial, received 2 cycles then stopped and remains with SD 5.1 years later.

**Table 4:** Best overall objective responses on protocol therapy according to dose level and sites of disease evaluable for response.

Dose level	No. of patients	Overall response		Soft tissue response		Bone marrow response		MIBG response	
		No. responses/total (%; 95% CI)	Response (no.)	No. responses/total (%; 95% CI)	Response (no.)	No. responses/total (%; 95% CI)	Response (no.)	No. responses/total (%; 95% CI)	Response (no.)
1	3	0/3	MR (1) SD (2)	1/1	PR (1)	0/1	SD (1)	0/3	SD (3)
2	3	0/3	MR (2) PD (1)	1/3	CR (1) SD (2)	2/3	CR (2) PD (1)	1/3	PR (1) SD (2)
2a	5^a^	1/5	PR (1) SD (1) PD (3)	0/4	SD (2) PD (2)	1/3	CR-MRD (1) SD (1) PD (1)	1/4	PR (1) SD (2) PD (1)
3a	6	1/6	PR (1) SD (3) PD (2)	1/4	CR (1) SD (2) PD (1)	1/4	CR (1) SD (3)	0/5	SD (3) PD (2)
4a	6	0/6	MR (1) SD (4) PD (1)	0/4	SD (3) PD (1)	0/1	SD (1)	1/5	CR (1) SD (4)
All dose levels	23	2/23 (8.7; 1.5–29.5)	PR (2) MR (4) SD (10) PD (7)	3/16^b^ (18.8; 5–46.3)	CR (2) PR (1) SD (9) PD (4)	4/12 (33.3; 11.3–64.6)	CR (3) CR-MRD (1) SD (6) PD (2)	3/20^c^ (15; 4–38.9)	CR (1) PR (2) SD (14) PD (3)

*CI* confidence interval, *CR* complete response, *CR-MRD* complete response-minimal residual disease, *MIBG* metaiodobenzylguanidine, *MR* minor response, *PD* progressive disease, *PR* partial response, *SD* stable disease.

^a^One patient withdrew before first disease evaluation for response.

^b^Among a total of 16 patients, 15 had soft tissue involvement at baseline, and 1 patient without soft tissue involvement at baseline had soft tissue disease progression at end of therapy.

^c^There are 23 patients with MIBG avid tumor at baseline, among which 20 were evaluated for MIBG response, 2 patients were not evaluated due to progressive disease at other sites at course 1, and 1 patient withdrew.

## Discussion

Polyamine enzymes are coordinately regulated by Myc to provide the essential intratumoral polyamines that support tumor progression [12]. As such, polyamine homeostasis is proposed as a targetable oncogenic output downstream of Myc in neuroblastoma [11, 33]. DFMO is a covalent inhibitor of ornithine decarboxylase (Odc), the rate-limiting enzyme in polyamine synthesis, so we investigated dose-esclated DFMO in combination with celecoxib and chemotherapy in this Phase 1 trial. Preclinical studies across neuroblastoma mouse models demonstrate anti-tumor activity and extended survival for mice taking ≥1% DFMO ad libitum [9, 10, 12, 19]. In these studies, average DFMO intake is ~2.1 mg/g-body weight per day based on water intake, which allometrically scales to a human equivalent dose of ~7 gm/m^2^/day DFMO [34]. This dose is significantly higher than those used in cancer chemoprevention trials [500-750 mg; [21, 35]], or in trials for neuroblastoma in first response after high-risk therapy completion (1500 mg/m^2^/d; [36]). Odc is highly expressed in neuroblastoma and has a short half-life with rapid proteolytic turnover, so despite DFMO being an irreversible inhibitor, higher DFMO exposures are required for clinically relevant Odc inhibition. Indeed, the extent to which polyamines are depleted in tumor cells may be pivotal since preclinical studies show markedly enhanced anti-tumor activity combining DFMO with drugs that increase polyamine export (celecoxib [12, 19]), inhibit uptake (AMXT1501 [12]), or inhibit downstream polyamine enzymes like SAM-decarboxylase (SAM486 [19]).

Here, we establish the tolerability of DFMO given at high doses combined with celecoxib and cyclophosphamide/topotecan chemotherapy, a common regimen for relapsed high-risk neuroblastoma [13, 37]. The MTD and recommended Phase 2 dose of DFMO with this regimen is 6750 mg/m^2^/day. The adult recommended Phase 2 dose was defined as 9000 mg/m^2^/day when combined with chemotherapy [32], yet DFMO dose intensity is higher in our trial as DFMO is given 21 of 28 days, as compared with 14 of 28 days in the adult setting. Prior adult carcinoma trials have not shown consistent anti-tumor activity but were not designed for *MYC*-driven tumors or supported by activity in relevant preclinical models. While hematopoietic toxicities were common in this heavily pre-treated neuroblastoma population at doses studied herein, no patient required chemotherapy dose modifications and the therapy was well tolerated overall, with infrequent dose reductions and 5 children completing 1-year of therapy. Further, only one patient had ototoxicity on this schedule that included a 7-day DFMO break (which was reversible and did not recur after resuming DFMO) despite prior cisplatin exposure and high ototoxic risk in this population [38].

A prior Phase 1 trial for relapsed neuroblastoma studied 18 children treated with oral etoposide and DFMO at lower doses from 1000 to 3000 mg/m^2^/day. One PR was seen (MIBG response) and this was at the 3000 mg/m^2^/day dose [24]. Here we escalated DFMO to higher doses and show a 9% objective response rate and 26% CR + PR + MR rate in heavily pre-treated individuals. The 2-year PFS and OS for the entire cohort was 29.5% and 58.3%, respectively, in this Phase 1 trial (Fig. 2). Notably, three patients (12.5%) remain progression free at >4 years in the absence of receiving additional therapy. All were diagnosed at <5 years of age and 1 has a *MYCN* amplified tumor, so these cases are not likely to represent *ATRX* mutated indolent neuroblastoma, common in older patients [39, 40]. Responses were seen in patients with *MYCN* amplified and non-amplified tumors. Both functional *ODC1* SNP risk alleles, G316 (rs2302615) [41] and T263 (rs2302616), were overrepresented in our relapsed/refractory patient cohort compared with population data though our study is underpowered to assess correlation with response or toxicity. In patients treated at the MTD of 6750 mg/m^2^/day, the mean trough DFMO concentration was >70 µmol/L, above that obtained in *TH-MYCN* mice treated with 1% DFMO in preclinical studies showing anti-tumor efficacy.

DFMO shows synergy with immunotherapy in preclinical models [42, 43] and may modify the tumor immune environment through effects on arginine availability [44] and immune effector cell functions [45, 46]. Given the tolerability of high-dose DFMO demonstrated on this trial, and the robust anti-tumor activity shown for irinotecan/temozolomide combined with dinutuximab and GM-CSF for relapsed neuroblastoma patients [47], an ongoing Children’s Oncology Group Phase 2 trial randomizes patients to receive this chemoimmuntherapy with or without the addition of DFMO at 6750 mg/m^2^/day (NCT03794349).

A limitation of this study is that tumoral polyamines were not measured either pre-therapy or post-therapy, as biopsy at those timepoints was not required on the study. Therefore, polyamine-directed bioactivity of the regimen is not directly assessed. Still, post-relapse PFS, recommended as an optimal primary endpoint for early phase neuroblastoma trials [48], is comparable to both real-world data with the COG chemoimmunotherapy regimem in multiply relapsed patients [2-year PFS 28%; [49]] and GD2-directed chimeric antigen receptor therapy (GD2-CART01 2-year PFS 27%; [50]). Polyamines can also be imported from the tumor microenvironment to rescue polyamine homeostasis in DFMO-treated tumors [8, 9]. AMXT1501 is a polyamine transport inhibitor that synergizes with DFMO in preclinical neural tumor models [12, 51] and is currently in human Phase 1/2 testing combined with DFMO (NCT03536728). An international trial to test DFMO and AMXT1501 (to augment tumoral polyamine depletion) with chemoimmunotherapy in children with relapsed neuroblastoma is under development. In conclusion, high-dose DFMO, celecoxib, and cyclophosphamide/topotecan is a tolerable and active regimen in patients with refractory or relapsed neuroblastoma, and efforts to optimize polyamine depletion with high-dose DFMO warrant further study.

### Supplementary information

Supplemental Table 1

## Data Availability

All data related to this trial are maintained within the NANT Clinical Trials database.
